# Prediction of HIV-1 virus-host protein interactions using virus and host sequence motifs

**DOI:** 10.1186/1755-8794-2-27

**Published:** 2009-05-18

**Authors:** Perry Evans, William Dampier, Lyle Ungar, Aydin Tozeren

**Affiliations:** 1 Genomics and Computational Biology and Department of Computer and Information Science, University of Pennsylvania, Levine Hall, 3330 Walnut Street, Philadelphia, PA 19104, USA; 2 Center for Integrated Bioinformatics, School of Biomedical Engineering, Science and Health Systems, Drexel University, Bossone 714, 3120 Market Street, Philadelphia, PA 19104, USA

## Abstract

**Background:**

Host protein-protein interaction networks are altered by invading virus proteins, which create new interactions, and modify or destroy others. The resulting network topology favors excessive amounts of virus production in a stressed host cell network. Short linear peptide motifs common to both virus and host provide the basis for host network modification.

**Methods:**

We focused our host-pathogen study on the binding and competing interactions of HIV-1 and human proteins. We showed that peptide motifs conserved across 70% of HIV-1 subtype B and C samples occurred in similar positions on HIV-1 proteins, and we documented protein domains that interact with these conserved motifs. We predicted which human proteins may be targeted by HIV-1 by taking pairs of human proteins that may interact via a motif conserved in HIV-1 and the corresponding interacting protein domain.

**Results:**

Our predictions were enriched with host proteins known to interact with HIV-1 proteins ENV, NEF, and TAT (p-value < 4.26E-21). Cellular pathways statistically enriched for our predictions include the T cell receptor signaling, natural killer cell mediated cytotoxicity, cell cycle, and apoptosis pathways. Gene Ontology molecular function level 5 categories enriched with both predicted and confirmed HIV-1 targeted proteins included categories associated with phosphorylation events and adenyl ribonucleotide binding.

**Conclusion:**

A list of host proteins highly enriched with those targeted by HIV-1 proteins can be obtained by searching for host protein motifs along virus protein sequences. The resulting set of host proteins predicted to be targeted by virus proteins will become more accurate with better annotations of motifs and domains. Nevertheless, our study validates the role of linear binding motifs shared by virus and host proteins as an important part of the crosstalk between virus and host.

## Background

This study focused on the computational identification of host proteins targeted by an invading virus, using HIV-1 infection as a case study because extensive study at the molecular level has yielded nearly fifteen hundred experimentally determined HIV-1, human protein interactions, which are catalogued in the HIV-1, Human Protein Interaction Database [1,2]. Virus and cellular parasite proteins alter host interaction networks by competing with host proteins for binding in the host protein-protein interaction (PPI) network [3-5]. Knowledge of which host proteins interact with virus proteins is important for antiviral drug discovery and treatment optimization using existing drugs [6]. Experimental approaches for finding virus protein binding partners in the human proteome have proved challenging because nearly thirty thousand human proteins must be tested [7]. Computational approaches have helped by reducing the number of host proteins to verify experimentally.

Previous host-pathogen interaction prediction methods focused largely on finding PPIs between human and cellular parasite proteins. One recent method found the probability that two protein domains interact given the human PPI network, and used this probability to find the likelihood that pathogen and human proteins interact given their domain profiles [8]. Another method matched human and pathogen protein pairs to proteins known to form complexes, and then filtered these interaction candidates based on expression data from human and pathogen [9]. Translating these methods to interactions between HIV-1 and human proteins has been difficult because HIV-1 proteins have few domains and their structures are hard to find by comparative modeling. For instance, to find structures for the N-terminal and C-terminal domains of HIV-1 VIF, two different protein structures were required for comparative modeling [10].

In our study, we focus on protein interactions mediated by short eukaryotic linear motifs (ELMs) [11] on HIV-1 proteins and human protein counter domains (CDs) known to interact with these ELMs. We aim to obtain host protein sets enriched with known sets of virus targeted proteins based on ELM and CD associations. The potential functional roles of interactions mediated by ELMs and their CDs in viral infection have been addressed in a number of recent articles [12-14]. The HIV-1 literature contains at least ten examples of HIV-1, human PPIs that are directly associated with motif and domain presence. The motif/domain basis of such PPIs is not restricted to a single HIV-1 protein, but is widely distributed across the HIV-1 proteome, including HIV-1 NEF [7], ENV [15], TAT [16], REV [16], VIF [17], and VPU [18]. This experimental evidence is the motivation for systematically investigating the association of motif/domain pairs with PPIs between virus and host proteins. Although Tastan et al. [19] estimated a relatively weak link between binding motif/domain presence and the actual virus-host PPIs, their work was restricted to predicting direct binding between host and HIV-1 proteins. In this study, we set out to identify host proteins involved in direct interactions as well as those that compete with HIV-1 proteins for binding to their host targets. Moreover, the algorithm presented by Tastan et al. is based on supervised learning and training from known interactions between HIV-1 and human proteins. In their method, each potentially interacting protein pair is associated with a feature vector composed of parameters related to Gene Ontology (GO), global gene expression profiles, the human protein interactome, and protein domains and motifs. Ours is a hypothesis-based approach, and does not require *a priori *knowledge of virus-host interactions beyond what can be gathered from viral and host protein sequences. As such, it is directly applicable to identifying host protein sets enriched with virus targeted host proteins for a wide scope of infectious diseases. The extremely low p-values we calculated for the overlap between our predictions and experimentally verified HIV-1, host protein interactions indicate the potential value of our approach for deducing a first draft of the molecular vocabulary employed in less studied host-pathogen protein interactions.

## Methods

### Virus protein ELM annotation and conservation

We downloaded the 2007 versions of multiple protein alignments for 9 (ENV, GAG, NEF, POL, REV, VIF, VPR, TAT and VPU) HIV-1 translated open reading frames from the HIV-1 Sequence Database  and removed all sequences except those labeled as subtypes B or C. We focused on subtype B because it is most common in the industrialized world [20], and chose subtype C because it is most common globally [21]. We computationally cleaved the GAG alignment into CA, MA, NC, P1, P2, and P6 alignments, and cleaved the POL alignment into IN, PR, and RT alignments using [GenBank: NC_001802] as a reference. All proteins in the resulting 18 alignments were annotated with ELMs using the ELM resource, accessed December 2008 [11], using default settings except selecting human for the species field. Any protein lacking an ELM was removed from the study, leaving at least 70 sequences in each multiple alignment [see Additional file 1]. We considered an ELM to be conserved on an HIV-1 protein if it was present on more than 70% of the protein's multiple alignment. This cutoff was chosen for its stability. An increase of 5% additional conversation did not alter the number of conserved ELMs (data not shown). A total of 99 ELMs were found on at least one virus protein sequence. The conservation threshold removed 43 of these, leaving 56 total.

### Human protein ELM and CD annotation

The ELM resource lists CDs or proteins known to interact with ELMs. For each ELM conserved on a virus protein, we found the appropriate CDs and mapped them to PROSITE domains [22]. When the ELM resource listed a set of interacting proteins instead of CDs, we assumed that all proteins had a common unknown CD, and annotated them with that. We constructed a list of CDs and interacting proteins for each HIV-1 conserved ELM [see Additional file 2].

We annotated PROSITE domains and ELMs on the 9446 human protein sequences in the Human Protein Reference Database (HPRD) PPI network [23], and mapped these sequences to Entrez GeneIDs. PROSITE domains were annotated with the PROSITE scan tool (release 20.31) using the default parameters [24]. ELMs were determined by using the ELM resource, accessed August 2008, selecting the same settings used for the HIV-1 sequences. Any protein lacking a PROSITE domain, or not binding to a protein with a PROSITE domain (other than itself), was removed from the study, leaving 5954 proteins.

### Prediction of human proteins interacting with HIV-1 proteins

The prediction of *HHP*, the set of human proteins that might interact with HIV-1 proteins, was based on interactions mediated by ELMs and CDs. We built *HHP *from the union of two sets of human proteins, *H1 *and *H2*. *H1 *was the set of human proteins predicted to directly interact with one or more HIV-1 proteins via a human CD and a virus ELM. *H2 *was the set of human proteins whose interactions with proteins in *H1 *were potentially disrupted by competition with an HIV-1 protein. Here an *H1 *protein has a CD that it might use to interact with an ELM present on both *H2 *and HIV-1 proteins. For example, in the competition between an HIV-1 and *H2 *protein for phosphorylation by an *H1 *kinase, the *H1 *protein has a kinase CD and the competing proteins have ELMs for phosphorylation sites.

The *HHP *prediction algorithm was straightforward. For each virus protein, we looked at all interactions documented in HPRD that could be explained by an interaction between a virus protein's conserved ELM and a CD known to interact with that ELM, and added the protein with the CD to *H1 *and the protein with the ELM to *H2*. Human proteins are involved in multiple interactions, so *H1 *and *H2 *are not mutually exclusive. *HHP *for each virus protein was the union of the protein's *H1 *and *H2 *sets, and contains all host proteins that either bind to or compete with the virus protein. *HHP *has 2348 proteins involved in 23330 predicted HIV-1, human interactions.

### Validation using the HIV-1, Human Interaction Database

The HIV-1, Human Protein Interaction Database (accessed August 2008) has 3,950 interactions between 19 HIV-1 proteins and 1,439 human proteins. All interactions for ENV's 2 cleavage products, GP41 and GP120, were assigned to ENV. Interactions for GAG and POL products were shown separately as well as assigned to GAG or POL. We restricted the human proteins interacting with HIV-1 proteins to those belonging to the set of 5954 proteins that have PROSITE domains and appear in the HPRD network with at least one non-self edge. The HIV-1, human interactions are spread over 68 interaction types, such as "interacts with", "phosphorlates", and "upregulates". We considered all interaction types, both direct and indirect. For each HIV-1 protein, we removed an interaction type if it described less than six interactions. This resulted in a set of 1,687 verified interactions between 15 HIV-1 proteins and 887 human proteins, which we called *HHE*, and used to investigate the usefulness of *HH*P. We constructed a subset of *HHE*, *DHHE*, which had interaction types deemed to be direct by Tastan et al. [19]. *DHHE *was used to evaluate *H1*.

The statistics in this research focused on the comparison of our predicted set *HHP *and the experimental dataset *HHE *based on the overlap between the two sets, GO molecular function enrichment, and KEGG pathway enrichment. P-values for the overlap between *HHP *and *HHE *and their various subsets were calculated using the hypergeometric test in the R Project for Statistical Computing. P-values for GO and KEGG enrichment for a given protein set compared to a background set of 5954 proteins were found using Bonferroni corrected p-values from DAVID [25].

## Results

### Human ELMs were conserved on HIV-1 proteins

**Figure 1:**
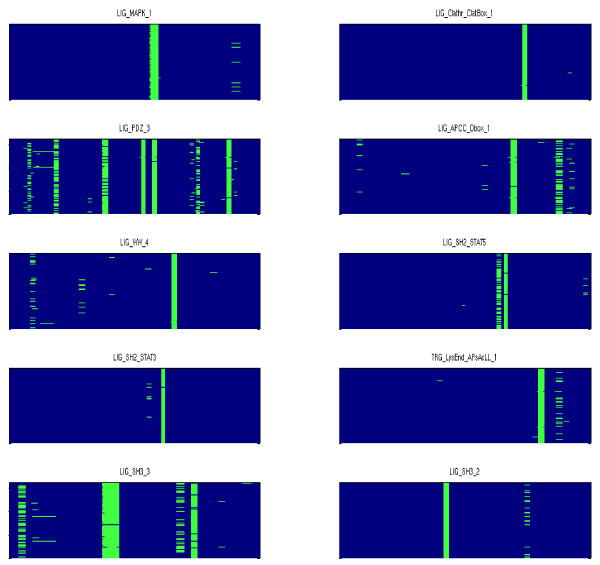
**ELM conservation on NEF**. ELMs were spatially conserved on alignments of HIV-1 proteins of subtypes B and C. Each box shows the annotations for one conserved ELM (present on more than 70% of protein instances) on the multiple alignment of NEF proteins. An ELM can be spatially conserved in multiple positions on the alignment, demonstrated by multiple sets of thick vertical lines in an ELM's box.

Figure 1 shows a subset of the conserved ELMs annotated on NEF's multiple alignment. It is clear from the figure that conserved ELMs occur in roughly the same position on each aligned protein. Our computations showed that this was true for all conserved ELMs on all HIV-1 proteins. Noting that HIV-1 is a virus with high mutation rate, these results support the assertion that conserved ELMs are essential for viral replication within the host cell [14]. ELM annotation in eukaryotic proteomes is not yet complete. Multiple computational strategies have been employed for the discovery of additional ELMs involved in protein interactions and post-translational modifications [26,27]. It is possible that HIV-1 proteins have additional conserved ELMs that have not yet been identified.

**Figure 2:**
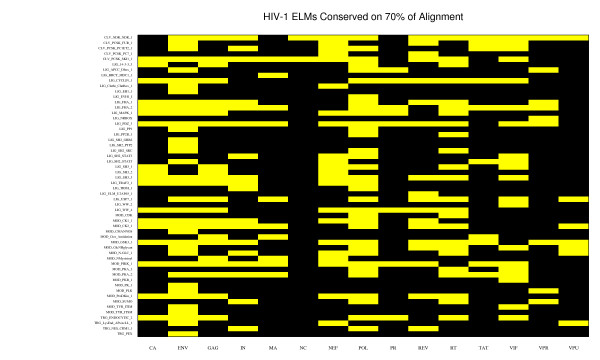
**Conserved ELMs on HIV-1 proteins**. Of 133 ELMs scanned, only 56 were conserved ELMs (present on more than 70% of an HIV-1 protein's alignment). Yellow boxes indicate conservation of an ELM above 70% for a virus protein. All HIV-1 proteins shown had at least one conserved ELM.

Conserved ELMs are shown for each HIV-1 protein in Figure 2. Overall, 56 of the 133 ELMs in the ELM resource were conserved on some HIV-1 protein. Some of the conserved ELMs, like the SH3 ligand sites on NEF, have been experimentally verified as binding sites for human proteins [28]. We found that conserved ELMs could occur frequently on human proteins. ELM LIG_PDZ_3 was seen on 90% of human proteins. Other ELMs, like LIG_EH1_1, appeared on only a few human proteins [see Additional file 2].

### *HHP *and *HHE *occupy the same KEGG pathways and share GO terms

**Figure 3:**
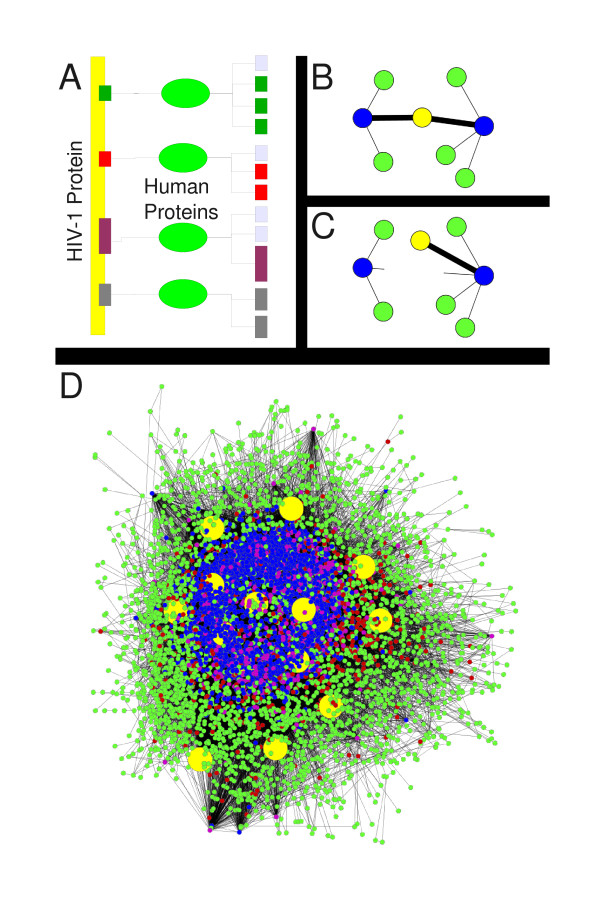
**Network diagrams for HIV1, host protein interactions**. (A) The scheme for identifying *HHP*. Rectangular blocks represent ELMs and ellipses represent their CDs. (B) An HIV-1 protein (yellow) alters the human PPI network by creating a new path between proteins (blue). (C) An HIV-1 protein breaks a path between two human proteins (blue) by competing for binding [3-5]. (D) Interactions between HIV-1 and human proteins, as predicted by *HHP *and *HHE *using HPRD. Nodes are proteins and edges represent a protein-protein interaction. Yellow nodes represent HIV-1 proteins. Purple nodes represent the overlap between *HHP *and *HHE*. Blue and red nodes represent proteins specific to *HHP *and *HHE*, respectively, while green nodes are not involved in infection.

**Figure 4:**
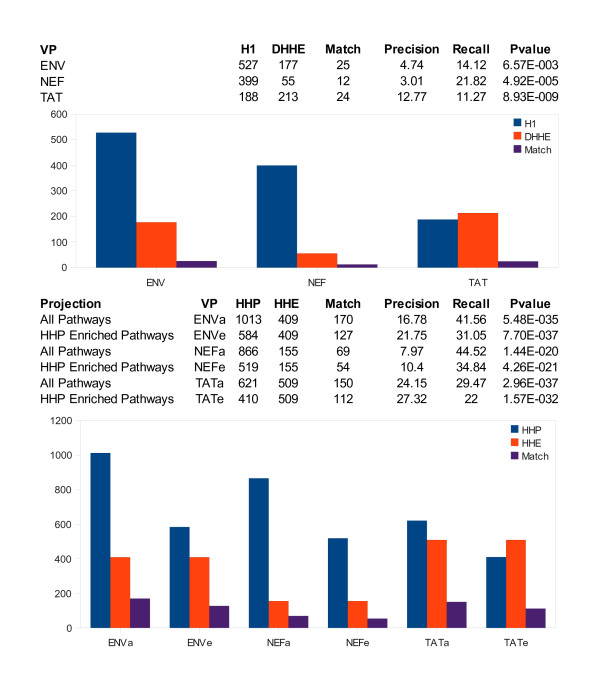
**Comparison of *H1 *and *DHHE *and *HHP *and *HHE *for KEGG proteins**. The upper half of the figure compares direct predictions (*H1*) with experimentally verified direct interactions (*DHHE*) for ENV, NEF and TAT. The p-values indicated a significant overlap for all protein sets. The bottom half of the figure compares *HHP *and *HHE *for the three HIV-1 proteins when *HHP *was restricted to genes in all KEGG pathways (ENVa, NEFa, TATa), and KEGG pathways enriched (p-value < 0.01, see Methods) with *HHP *(ENVe, NEFe, TATe). The intersection between *HHP *and *HHE *was significant for both projections, but slightly more significant for enriched pathways for ENV and NEF. P-values were calculated as described in Methods.

*HHE *contains 887 host proteins known to interact with one or more HIV-1 proteins. The dataset is noisy as it includes results from multiple laboratories and varying methodologies, some of which might not have been sensitive enough to identify direct binding partners within a collection (complex, aggregate) of proteins. Nonetheless, *HHE *was appropriate for the task of assessing *HHP*. The HPRD network of the 5954 proteins in the study is shown in Figure 3D with yellow HIV-1 proteins connected to proteins in *HHP *(blue) and *HHE *(red). Proteins in both sets are purple, while all other proteins are green. As seen in the figure, *HHP *was larger in size than the corresponding *HHE *for an HIV-1 protein. Proteins in *H2 *dominated the overlap between *HHP *and *HHE*, and many of the proteins in *H1 *were also found in *H2*. We investigated the usefulness of *H1 *by comparing it with *DHHE*, the subset of *HHE *with only direct interactions, and found that there were some virus proteins for which there was significant overlap between *H1 *and *DHHE *[see Additional file 3]. The upper half of Figure 4 shows the overlap p-values and sizes of *DHHE *and *H1 *for ENV, NEF, and TAT, which were the HIV-1 proteins with the largest *HHE *sets.

We found KEGG pathways enriched with proteins from each virus protein's *HHP *set (p-value < 0.01, see Methods). Shown in the lower half of Figure 4 are bar graphs demonstrating the intersection of *HHP *in KEGG (or *HHP *found in enriched pathways) and *HHE *for ENV, NEF, and TAT. Our model predicted 584, 519, and 410 proteins will interact with ENV, NEF, and TAT, respectively, and matched 127 of 409, 54 of 155, and 112 of 509 experimentally verified interactions. The p-values indicated a statistically significant match between predicted and experimental sets for ENV, NEF, and TAT when using both direct predictions (*H1*), and direct predictions in addition to competing predictions (*HHP*). However, the p-values in Figure 4 showed that the overlap between predicted and experimental data was weaker for *H1 *and *DHHE *than for *HHP *and *HHE*.

The intersection between *HHP *and *HHE *for HPRD proteins became more significant as we took the projections of these sets onto the set of human proteins in KEGG pathways [see Additional file 4]. The significance improved further when ENV and NEF *HHP *were further restricted to genes in KEGG pathways that were statistically enriched with *HHP *(p-value < 0.01). One potential contributor to such low p-values is that host proteins in KEGG pathways are among the most studied, and therefore their interactions with HIV-1 proteins would have been investigated earlier than the poorly studied host proteins. Nevertheless, the correspondence between statistically enriched *HHP *and *HHE *KEGG pathways (Table 1, p-value < 0.01) and the enriched GO molecular function level 5 categories (Table 2, p-value < 0.01), suggested the co-localization of *HHP *and *HHE *in the host proteome.

**Table 1:** KEGG Pathway Enrichment

	ENV HHP	ENV HHE	NEF HHP	NEF HHE	TAT HHP	TAT HHE
AML	1.97E-05	2.68E-05	2.07E-05	8.55E-05	2.66E-08	3.08E-04
Adherens junction	2.15E-09	NA	8.21E-11	NA	6.74E-06	NA
Apoptosis	4.58E-04	1.43E-13	5.18E-04	6.54E-04	3.91E-03	3.88E-13
B cell receptor signaling pathway	4.86E-06	8.73E-10	2.88E-04	2.90E-04	1.25E-09	5.57E-06
Cell cycle	NA	NA	NA	NA	2.88E-04	1.90E-01
CML	7.21E-05	2.23E-05	1.17E-06	2.46E-05	1.01E-09	7.53E-07
Colorectal cancer	1.07E-05	3.18E-04	4.68E-08	3.31E-03	4.50E-04	1.42E-03
Endometrial cancer	6.03E-04	1.62E-02	1.66E-04	2.10E-03	5.03E-07	3.83E-04
Epithelial cell signaling in H. pylori infection	2.29E-04	3.84E-06	2.07E-06	2.83E-05	NA	NA
ErbB signaling	2.27E-10	5.70E-04	1.70E-12	9.22E-04	1.53E-12	1.66E-05
Fc epsilon RI signaling pathway	8.43E-04	6.23E-21	2.14E-05	2.20E-07	9.62E-05	1.52E-04
Focal adhesion	2.82E-06	2.30E-03	2.31E-07	6.90E-02	5.28E-08	3.36E-09
Gap junction	1.90E-04	1.26E-04	NA	NA	1.12E-04	7.18E-10
Glioma	1.50E-04	1.24E-05	4.99E-06	6.02E-03	1.56E-07	6.79E-11
Insulin signaling	1.53E-07	8.84E-02	1.73E-04	3.50E-01	2.91E-07	7.35E-02
Jak-STAT signaling	4.08E-08	2.15E-04	4.09E-09	1.28E-01	2.32E-17	4.91E-03
Leukocyte transendothelial migration	1.94E-07	2.21E-08	1.17E-08	6.36E-01	3.28E-05	1.45E-01
Long-term potentiation	6.79E-05	2.20E-02	NA	NA	9.04E-03	2.38E-10
MAPK signaling	5.19E-08	6.32E-04	1.58E-09	3.27E-03	1.18E-03	5.15E-01
NK cell mediated cytotoxicity	NA	NA	NA	NA	9.50E-06	5.31E-15
Non-small cell lung cancer	4.28E-05	1.25E-04	1.26E-05	1.67E-03	7.55E-06	1.45E-06
Pancreatic cancer	1.26E-04	5.54E-07	1.10E-05	2.50E-06	1.03E-05	8.15E-08
Pathogenic E. coli infection – EHEC	3.77E-03	1.00E+00	2.94E-03	NA	9.74E-03	3.25E-01
Phosphatidylinositol signaling system	1.36E-03	1.72E-04	2.37E-03	NA	2.19E-05	9.74E-06
Prostate cancer	1.90E-04	1.26E-04	6.26E-06	6.56E-05	5.46E-09	1.11E-07
Regulation of actin cytoskeleton	4.30E-03	6.02E-01	1.73E-03	8.79E-01	2.66E-03	7.65E-01
Small cell lung cancer	1.94E-03	3.71E-10	8.42E-05	4.25E-02	1.12E-04	4.09E-14
T cell receptor signaling pathway	NA	NA	NA	NA	1.56E-06	1.35E-11
Tight junction	1.24E-03	1.00E+00	5.29E-04	NA	NA	NA
Toll-like receptor signaling pathway	5.16E-03	2.04E-14	5.37E-05	2.04E-14	NA	NA
Type II diabetes mellitus	NA	NA	NA	NA	3.47E-03	5.95E-01
VEGF signaling	3.23E-03	4.89E-15	6.79E-03	8.82E-03	1.88E-05	4.07E-12

KEGG Pathways enriched (p-value < 0.01, see Methods) in *HHP *for HIV-1 ENV, NEF, and TAT. *HHE *enrichment is also indicated.

**Table 2:** Gene Ontology Enrichment

	ENV		NEF		TAT	
	HHP	HHE	HHP	HHE	HHP	HHE
adenyl ribonucleotide binding	9.9E-10	9.9E-10	4.8E-10	4.8E-10	7.8E-12	7.8E-12
inositol or phosphatidylinositol kinase activity	6.6E-06	6.6E-06	1.6E-06	1.6E-06	7.7E-06	7.7E-06
interleukin receptor activity	3.4E-07	3.4E-07	4.7E-08	4.7E-08	6.0E-08	6.0E-08
lipid kinase activity	1.4E-04	1.4E-04	4.9E-05	4.9E-05	1.3E-05	1.3E-05
MAP kinase activity	1.4E-04	1.4E-04	4.1E-05	4.1E-05	NA	NA
MAP kinase kinase kinase activity	2.3E-03	2.3E-03	7.5E-04	7.5E-04	NA	NA
phosphoric monoester hydrolase activity	4.9E-07	4.9E-07	1.9E-03	1.9E-03	NA	NA
protein kinase activity	7.9E-32	7.9E-32	1.6E-28	1.6E-28	6.6E-33	6.6E-33
protein kinase binding	1.1E-05	1.1E-05	1.7E-04	1.7E-04	2.0E-07	2.0E-07

GO molecular function level 5 categories statistically enriched (p-value < 0.01) by *HHP *for ENV, NEF, and TAT. *HHE *enrichment is also indicated.

The KEGG pathways statistically enriched for ENV, NEF, and TAT interacting proteins (experimental as well as computational) included immune system pathways such as T cell and B cell receptor signaling pathways, apoptosis, focal adhesion, and toll-like receptor signaling pathways (Table 1). Gene expression data before and after HIV-1 infection of macrophages also showed apoptosis and MAPK signaling pathways as statistically enriched [29], as predicted here. Microarray results did not show cell cycle and toll-like receptor pathways as highly activated in HIV-1 activated macrophages, although the toll-like receptor pathway was highly enriched with known HIV-1 targeted proteins (Table 1). Also statistically enriched were disease pathways such as the colorectal cancer, leukemia, and lung cancer pathways that have been shown to have high incidence of occurrence in HIV-1 infected individuals [30]. Other disease pathways predicted by our analysis included those previously associated with HIV-1 infection: *H. pylori *infection [31], *E. coli *infection [32], and type II diabetes [33]. These observations indicated the promise of our method in predicting activated disease pathways based on viral sequence. Post-translational modification appeared to be an important element of HIV-1 cellular network hijacking. As shown in Table 2, protein kinase activity and protein kinase binding were highly statistically enriched both in *HHP *and *HHE*, suggesting the importance of altered phosphorylation events in the reorientation of the host cell PPI network towards virus survival and replication [29]. The HIV-1 activated GO categories listed in Table 2 are associated with signal transduction processes in the KEGG pathways presented in Table 1.

**Figure 5:**
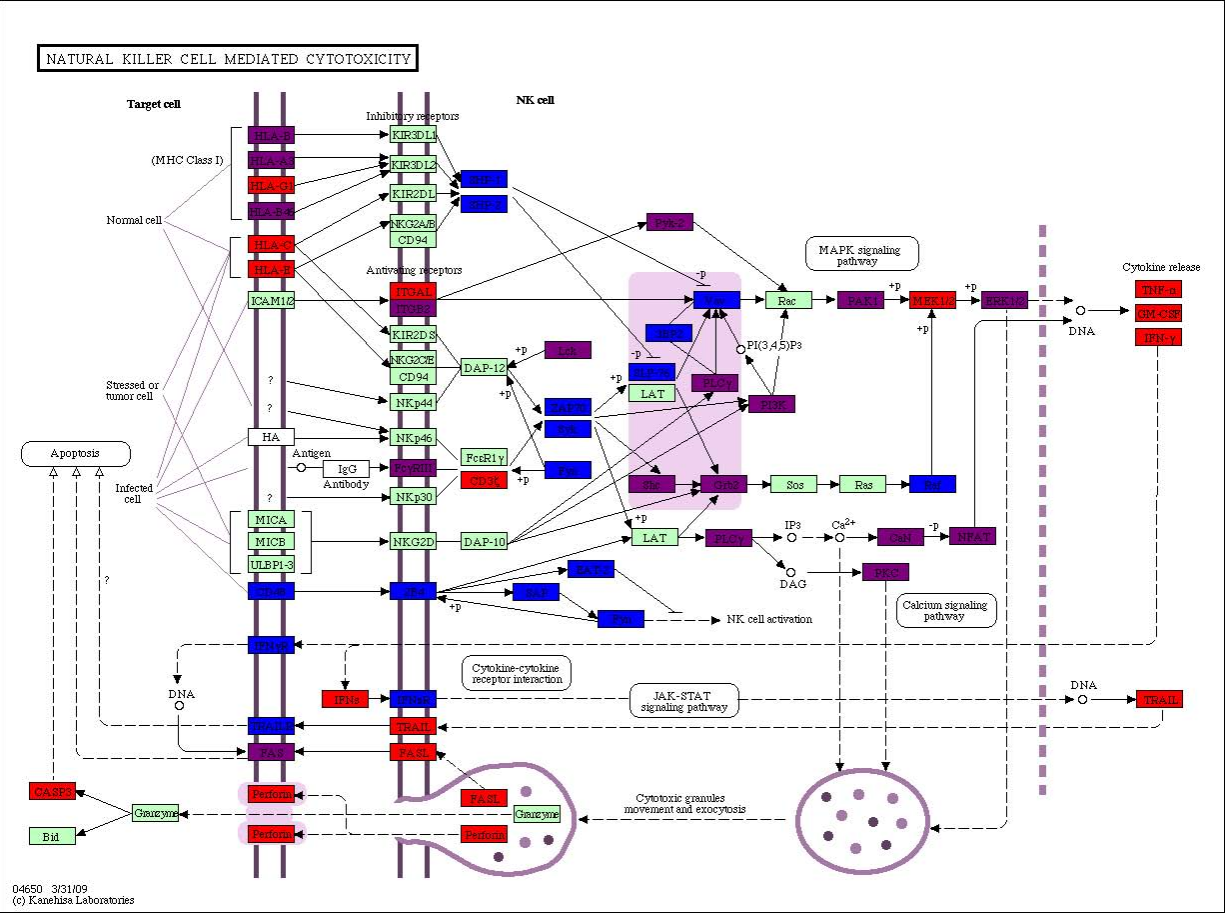
**HIV-1 TAT natural killer cell mediated cytotoxicity**. The KEGG natural killer cell mediated cytotoxicity pathway is colored for TAT *HHP *(blue) and *HHE *(red), and their overlap (purple). Green boxes have proteins not involved in infection, while white boxes do not have human proteins.

The positions of predicted and matched HIV-1 targeted proteins along KEGG pathways allowed us to assess the extent of matching between computational and experimental prediction based on cell-compartment identity. Figure 5 shows the match (purple) between predicted (blue) and experimentally determined (red) host proteins targeted by HIV-1 TAT along the natural killer cell mediated cytotoxicity pathway. Our predictions were on target on the cell membrane for HLA-B, HLA-A3, HLA-B45, and FAS, but we missed Perforin, HLA-C, HLA-E, and HLA-G1. The figure also shows a good match for DNA transcription factors targeted by HIV-1. The green boxes in the figure correspond to host proteins with apparently no direct interaction with TAT.

**Figure 6:**
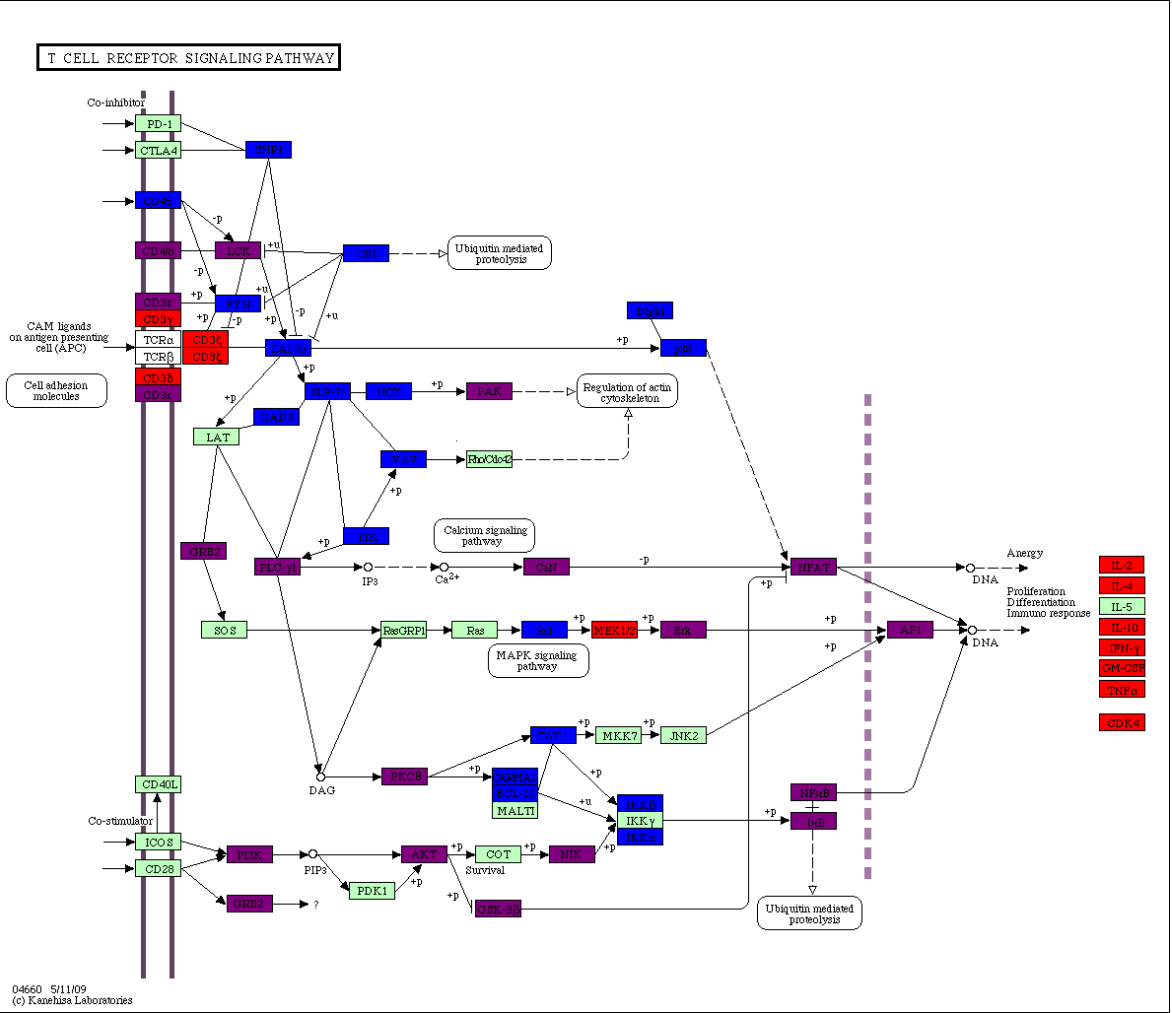
**HIV-1 TAT T cell receptor signaling pathway**. The KEGG T cell receptor signaling pathway is colored for TAT *HHP *(blue) and *HHE *(red), and their overlap (purple). Green boxes have proteins not involved in infection, while white boxes do not have human proteins.

The cytokines shown in red at the right hand side of the KEGG diagram in Figure 5 would not be expected to appear in our predicted list. They belong to *HHE*, but their interactions with virus proteins are probably not direct, but via transcriptional regulation. The T cell receptor signaling pathway in Figure 6 indicates a high degree of matching (purple) along the cell membrane and on transcription factors between TAT targeted host proteins (red) and our corresponding predictions (blue). The abundance of predicted host proteins in the pathway with no matching experimental result suggests new PPI interaction studies for HIV-1 as well as further refinement of our computational method.

**Figure 7:**
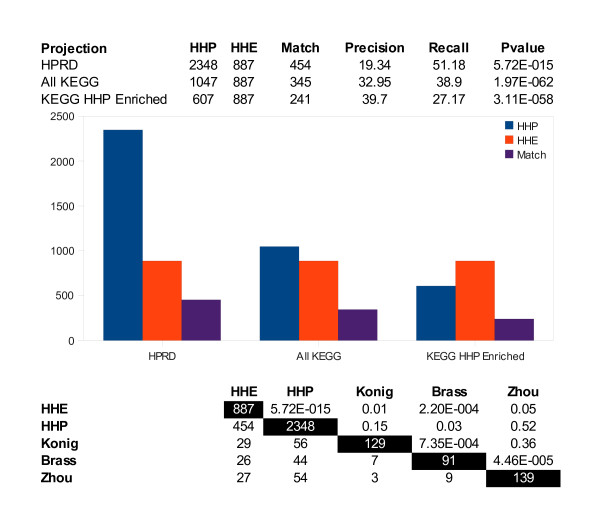
**Comparison of combined *HHP *and *HHE***. *HHP *and *HHE *are combined across all HIV-1 proteins. *HHP *performance is compared when restricting it to genes in HPRD, KEGG, and KEGG pathways enriched in *HHP *(p-value < .01, see Methods). The lower table compares *HHE*, *HHP*, and predictions from three siRNA screens. The darkened diagonal holds the sizes of all sets. The overlap between sets is below the diagonal, while p-values for these overlaps are above (see Methods).

Figure 7 shows a combined view of *HHP *and *HHE*, made by aggregating *HHP *and *HHE *for all virus proteins. When we looked at *HHP *in KEGG, we had 1047 host proteins, and 345 of these had already been shown to be interacting with at least one HIV-1 protein. The match between computational prediction and experimental data in this case led to a p-value of 1.97 E-62. One reason for the small p-value is that a host protein was considered to be interacting with HIV-1 even if the protein interacted with an HIV-1 protein other than the one that was experimentally verified. Nevertheless, this virus protein insensitive set is meaningful, as it provides a first estimate of HIV-1 targeted host proteins.

### ELM Modules did not perform better than ELMs

**Figure 8:**
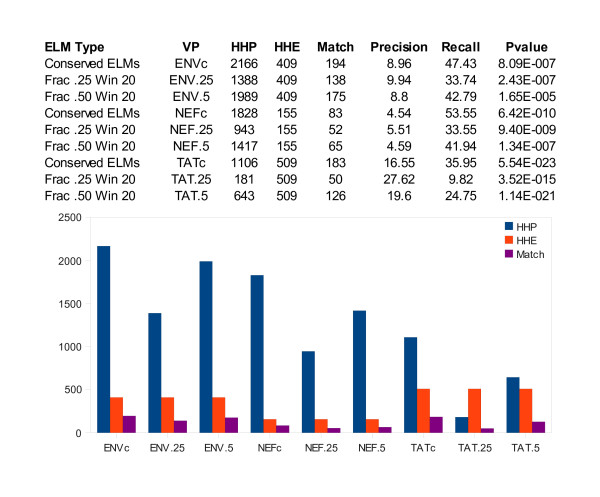
**Comparison of *HHP *and *HHE *for HPRD proteins**. HIV-1 proteins ENV, NEF, and TAT had significant overlap between *HHP *and *HHE*. This was true when we looked at all conserved ELMs or ELM pairs occurring below some fraction (Frac) in the human proteome. P-values were calculated as described in Methods.

Next we asked if restricting our analysis to ELMs and ELM pairs with low frequencies of occurrence in the host proteome would yield a better *HHP*, hypothesizing that frequent ELMs were causing false positives. In an effort to reduce the frequency of ELM occurrence, we looked for ELM modules, defined as two different ELMs occurring in a 20 residue window. We identified ELM modules conserved on more than 70% of each HIV-1 protein's multiple alignment, as we did for ELMs. We found the fraction of human proteins with each ELM or ELM module, and chose two frequency cutoffs, 0.25 and 0.50, to restrict the ELMs and ELM modules on virus proteins to those that were infrequent on human sequences. Any ELM or ELM module with a human frequency above the cutoff was not used to predict interactions. Figure 8 shows the results for ENV, NEF, ad TAT, comparing the use of all conserved ELMs to using frequency (fraction) cutoffs for conserved ELMs and ELM modules. The results indicated that such restrictions on ELMs helped results for ENV, but not for NEF and TAT. For NEF and TAT, ELM restrictions yielded smaller *HHP*, but the overlap between *HHP *and *HHE *was also reduced.

## Discussion

The rapid sequencing of viral genomes with next generation sequencing technology [34] makes it possible to link clinical parameters of viral infection to sequence motifs. The task of identifying host proteins targeted by a virus is worthwhile because such proteins may become drug targets to fight infection [6]. Experimental studies for determining virus targeted proteins are expensive and highly challenging [14]. Such efforts, although large-scale, have produced incomplete results for even well studied viruses like HIV-1 [6,35,36]. In this study, we used a systems approach to identify host protein subsets enriched by virus targeted proteins. Our method was based on the identification of host motifs on virus sequences. We used the *a priori *knowledge in the ELM resource to identify the counter domains associated with these motifs and information from the human interactome to focus on host protein interaction pairs with appropriate motif/domain links. KEGG pathways and the GO molecular functions were used to provide biological context to our findings.

The sets of host proteins we predicted as targeted by a given HIV-1 protein in KEGG pathways were highly statistically enriched with host proteins known to interact with the same HIV-1 protein (Figure 4). For example, the match between our predictions and the interactions for HIV-1 NEF in the HIV-1, Human Protein Interaction Database corresponded to a p-value of 4.26 E-21 in KEGG pathways enriched in our predicted set. After combining our predictions for all HIV-1 proteins, we had 607 proteins in *HHP *enriched KEGG pathways, and of these we matched 241 in the set of 877 experimentally verified proteins with a p-value of 3.11 E-58 (Figure 7). Our predictions were not nearly an exact match for experimental data, but our list was highly enriched with HIV-1 targeted host proteins. Given that *HHP *in KEGG pathways is about half as large as all *HHP*, and has a stronger overlap with *HHE*, experimentalists should begin verification with this set.

In addition to the binding/interaction research compiled in the HIV-1, Human Protein Interaction Database, recent experimental studies based on genome-wide siRNA screens have brought additional light to host-pathogen interactions that facilitate HIV-1 replication [6,35,36]. Three studies produced smaller lists of host proteins than the list in the HIV-1, Human Protein Interaction Database. The lower matrix in Figure 7 shows the five-way comparison of HIV-1 targeted protein lists: *HHE*, *HHP*, and the three screens. The table indicated the extent of discrepancy between lists, as well as the statistical significance of the matches between them. Our predictions matched *HHE *with the lowest p-value, and the genome-wide study lists generally matched each other better than the interaction studies. The list of 280 genes presented as host cellular factors required for HIV-1 replication by Brass et al. had 13 genes in common with the list of 295 genes deemed necessary by Konig et al. for regulation of early stage HIV-1 replication, and shared 10 genes with the 311 genes given in the Zhou study. When these proteins were projected into HPRD, the matches led to p-values of 7.35 E-4 and 4.46 E-5. Although the match was significant, there was still a discrepancy between the results. This mismatch may be attributed to the differences in the analysis and experimental methodologies used. Our predictions matched 56 of the 129 HPRD proteins presented by Konig et al. with a p-value of 0.15, 44 of the 91 HPRD proteins in the list by Brass et al. with a p-value of 0.03, and 54 of the 139 HPRD proteins given by Zhou et al. with a p-value of 0.52. These results indicated the challenges faced by experimental studies trying to uncover the grammar of HIV-1, host interactions.

Although our study produced host protein sets statistically enriched with proteins known to be targeted by HIV-1, mismatches between our predictions and experimental data cannot be ignored. It is possible that host-virus interactions employ a grammar that is much more complex than the short linear motif/counter domain interactions assumed in this study. The molecular vocabulary of PPIs is simply not well understood even for proteins belonging to the same species. However, one common mode of interaction is the binding of a linear binding motif on one protein to a domain on another protein [37]. A central hypothesis in the discovery of the linear binding motifs mediating protein interactions has been that proteins with a common interacting partner, such as protein kinases, share a common feature in the form of a motif [38]. Some of the linear binding motifs in the ELM resource have been shown to bind directly to sites at opposing counter domains listed in databases such as PROSITE and Pfam [39]. However, for approximately 30% of the PPI interactions listed in HPRD database, interacting proteins possess none of the already annotated domains. Thus, a model based on known motif/domain interactions would not be able to capture all of the known interactions in the host, let alone those between virus and host.

Another important cause of the discrepancy between our predictions and experimental data might have been the poor annotation of known motifs and counter domains used in this study [40]. Recent studies of domain-motif interactions indicated that the annotation signatures are more specific than those presented in ELM and PROSITE. This was found to be true for the HIV-1 interacting PDZ domain [12], SH3 domain [13] and others [14]. Emerging motif finding tools such as DILIMOT [41], SLIMFinder [42], and D-STAR [43] will help researchers improve the specificity of the motifs that mediate host-virus interactions. Still, the list of host proteins we have provided [see Additional file 5] comprises a candidate set for genome-wide studies of the regulation of HIV-1 replication and infection.

We focused on HIV-1 infection in this study because we desired to assess the effectiveness of our computational approach by comparing our predictions with large-scale experimental data. Our results provided a rationale for applying our method to predict virus-human interactions for sequenced viruses. A systems approach to predicting host-pathogen interactions will at least be partially based on the sequence motifs of interacting genome/proteomes. The present study illustrated the importance of ELMs in the molecular cross talk between host and virus and opened the door for more extensive experimental and computational studies of host-virus interactions.

## Conclusion

In this study, we described a bioinformatics model to investigate the crosstalk between the HIV-1 and human proteins. Our method used multiple sequence alignments of HIV-1 proteins, and three datasets related to the host: decoded sequences of the host proteins, *a priori *knowledge of experimentally observed protein-protein interactions within the host proteome, and interactions between short linear peptide motifs and protein domains. The output of the model was a list of host proteins that may interact with specific HIV-1 proteins using specific sites. This list can be used to draft a connectivity map between virus and host, and to determine a set of protein interaction pathways that are significantly enhanced by host proteins predicted to be targeted by HIV-1.

The model was based on the assumption that virus proteins interact with host proteins though a set of conserved linear sequence motifs present in the host proteome. The conserved spatial organization of these motifs on the rapidly evolving HIV-1 proteome supported the assertion that short linear motifs play critical roles in interactions with the host network. The model's predictions led to host protein sets that are crowded by known HIV-1 targeted proteins. This statistical enrichment was particularly high along cellular pathways modulated by HIV-1. The model's predictions were also consistent with experimental data showing phosphorylation events as key targets of HIV-1 when redirecting cell protein networks toward the goal of virus replication.

The methodology applied here for HIV-1, host protein interactions is applicable to any viruses with multiple sequence alignments and hosts with known interactomes. Therefore, our approach has potential use in the identification of host proteins targeted by emerging and/or understudied viruses. The resulting list will be useful for selecting optimal drug therapies and discovering new antivirus drugs. The systems approach presented here for predicting host-virus protein interactions will benefit from ongoing research on the more specific annotations of short linear motifs and domains involved in protein-protein interactions.

## Competing interests

The authors declare that they have no competing interests.

## Authors' contributions

This study was conceived by PE and AT with significant input to intellectual content at all stages from WD and LU. PE executed the model operations in collaboration with WD. All authors worked on and approved the final manuscript.

## Pre-publication history

The pre-publication history for this paper can be accessed here:



## Supplementary Material

Additional file 1 **Number of sequences in HIV-1 protein alignments**. For each HIV-1 protein, we list the number of sequences used in the study. Click here for file

Additional file 2 **ELMs and CDs**. For each ELM-CD pair, the fraction of human proteins with each is listed along with the fraction of HPRD linked proteins that have the ELM-CD relation. Click here for file

Additional file 3 **Comparison of *H1 *and *DHHE *for each HIV-1 protein**. *H1 *and *DHHE *are compared for all HIV-1 proteins, giving the overlap (Match) between the two protein sets. P-values are calculated as the probability of matching Match genes or more when comparing *DHHE *and *H1 *drawn from the 5954 proteins in the study (see Methods). Click here for file

Additional file 4 **Comparison of *HHP *and *HHE *for KEGG proteins for each HIV-1 protein**. *HHP *and *HHE *are compared for all HIV-1 proteins when *HHP *is restricted to genes in all KEGG pathways, and KEGG pathways enriched (p-value < 0.01, see Methods) with *HHP*. P-values are calculated as the probability of matching Match genes or more when comparing *HHE *and *HHP *drawn from the 5954 proteins in the study (see Methods). Click here for file

Additional file 5 ***H1 *and *H2 *for each HIV-1 protein**. This table gives *HHP *as *H1*-*H2 *Entrez GeneID pairs for each HIV-1 protein. Proteins in *HHE *are marked with *, proteins in *HHP *KEGG enriched pathways are marked with +, and proteins in both are marked with **. Click here for file
